# The Differentiation and Regeneration Potential of ABCB5^+^ Mesenchymal Stem Cells: A Review and Clinical Perspectives

**DOI:** 10.3390/jcm14030660

**Published:** 2025-01-21

**Authors:** Zheng He, Vytaute Starkuviene, Michael Keese

**Affiliations:** 1 BioQuant, Heidelberg University, Im Neuenheimer Feld 267, 69120 Heidelberg, Germany; zheng.he@bioquant.uni-heidelberg.de; 2 European Center of Angioscience (ECAS), Medical Faculty Mannheim, Heidelberg University, Ludolf-Krehl-Straße 13-17, 68167 Mannheim, Germany; 3 Institute of Biosciences, Vilnius University Life Sciences Center, 10257 Vilnius, Lithuania; 4 Department of Vascular Surgery, Theresienkrankenhaus, Bassermannstraße 1, 68165 Mannheim, Germany

**Keywords:** ABCB5, mesenchymal stem cells, cell therapy, regeneration

## Abstract

Mesenchymal stem cells (MSCs) are a family of multipotent stem cells that show self-renewal under proliferation, multilineage differentiation, immunomodulation, and trophic function. Thus, these cells, such as adipose tissue-derived mesenchymal stem cells (ADSCs), bone marrow-derived MSCs (BM-MSCs), and umbilical cord-derived mesenchymal stem cells (UC-MSCs), carry great promise for novel clinical treatment options. However, the challenges associated with the isolation of MSCs and the instability of their in vitro expansion remain significant barriers to their clinical application. The plasma membrane-spanning P-glycoprotein ATP-binding cassette subfamily B member 5 positive MSCs (ABCB5^+^ MSCs) derived from human skin specimens offer a distinctive advantage over other MSCs. They can be easily extracted from the dermis and expanded. In culture, ABCB5^+^ MSCs demonstrate robust innate homeostasis and a classic trilineage differentiation. Additionally, their ability to modulate the recipients’ immune system highlights their potential for allogeneic applications in regenerative medicine. In this review, we primarily discuss the differentiation potential of ABCB5^+^ MSCs and their perspectives in regenerative medicine.

## 1. Introduction

Mesenchymal stem cells, as non-hematopoietic multipotent stem cells, are capable of differentiating into mesenchymal tissue lineages, such as adipocytes, osteocytes, chondrocytes, and endothelial cells [1,2]. These cells also regulate immune homeostasis by direct or indirect influence on immune cells [3]. Furthermore, MSCs exhibit an exocrine function, secreting growth factors, chemokines, interleukins, and extracellular matrix (ECM) molecules that are essential for the normal function of tissues and organs [4,5,6]. Initially identified in bone marrow [7], human MSCs have been derived from adipose tissue [8,9], skeletal muscle [10], placenta [11], umbilical cord blood [12], and nearly all other adult connective tissues. To date, MSCs have been shown to play vital roles in regeneration, metabolic homeostasis, immunomodulation, and trophic functions across various tissues [13].

While recent improvements in people’s quality of life has led to progress in medical technologies, the epidemiological burden of both chronic local and chronic systemic diseases has simultaneously increased rapidly. For many patients, there remains an unmet need for satisfactory treatment options. Since the first clinical therapeutic use of ex vivo-expanded MSCs for hematologic malignancies in 1995 [14], the potential of MSCs has been explored for a wide array of multiple clinical fields, such as in tissue and organ regeneration, immune modulation, cardiovascular disorders, and in cancer therapy (Table 1).

MSCs possess several characteristics that allow them to be employed in therapy, such as easy isolation, in vitro expansion, self-renewal ability, the potential for multilineage differentiation, trophic functions, and immunomodulatory effects [35]. However, in vitro morphological abnormalities -and the degeneration or disappearance of specific surface markers have been observed in bone marrow MSCs [36]. Similarly, genomic instability has been reported in UC-MSCs [37]. Consequently, MSCs may exhibit reduced proliferation and impaired differentiation, which can be attributed to factors such as the donor’s age, prolonged culture duration, suboptimal media supplements, or culture conditions [38]. Moreover, when only a small number of autologous MSCs can be obtained, the process of in vitro expansion can be time-consuming, potentially attenuating the treatment effect. While induced pluripotent stem cell (iPSC) technology offers a method to generate autologous MSCs in large quantities, it is limited by low reprogramming efficiency, tumorigenesis risks, high production costs, and regulatory challenges [39]. In contrast, ABCB5^+^ MSCs can be easily isolated from discarded skin tissue and expanded efficiently in vitro, offering a practical and scalable alternative for regenerative therapies.

## 2. Characteristics of ABCB5^+^ MSCs as a Novel Mesenchymal Stem Cell

ABCB5^+^ MSCs are a distinct subpopulation of dermal MSCs that express the specific surface marker ABCB5. In vivo, these cells are either confined to the peri-vascular endogenous niche, closely associated with CD31^+^ endothelial cells, or they can be found dispersed within the interfollicular dermis independent of hair follicles [40]. ABCB5^+^ MSCs can be isolated from enzymatically digested skin tissue by ABCB5 magnetic bead sorting.

**Figure 1:**
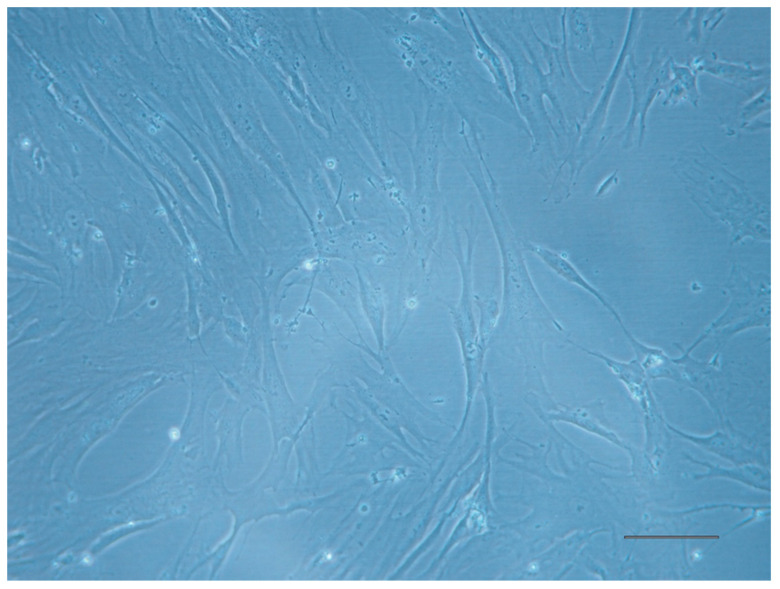
ABCB5^+^ MSCs. Cultured ABCB5^+^ MSCs are shown at passage 5, grown in Ham’s F10 Medium supplemented with 10% (*v*/*v*) FBS, 1% (*v*/*v*) penicillin/streptomycin (P/S), and 1% (*v*/*v*) L-glutamine (Scale bar: 100 µm).

These cells are plastic-adherent and exhibit a fibroblastoid, spindle-shape morphology similar to classic mesenchymal stem cells (Figure 1). When analyzed or sorted by flow cytometry, ABCB5^+^ MSCs express the core mesenchymal lineage markers CD90, CD105, and CD73 while lacking hematopoietic and lineage-specific markers CD34, CD14, CD20 and CD45 [40,41]. Thus, these cells meet the criteria for classification as a distinct mesenchymal stem population [42].

ABCB5 is a transmembrane P-glycoprotein that regulates the efflux of some substances, including chemotherapeutic drugs, and is therefore recognized as a multidrug resistance (MDR) transporter protein [43]. Additionally, ABCB5 is considered a cancer cell marker, mediating chemoresistance through drug transport [44]. This p-glycoprotein exhibits a selective tissue expression pattern across human physiological tissues and cancers and can also specifically identify a subpopulation of skin progenitor cells [41,45].

Skin-derived ABCB5^+^ MSCs can be easily and reliably isolated using a specific antibody directed against an extracellular-loop sequence of the ABCB5 molecule [45]. In vitro, cells can be expanded even on a large scale. Therefore, these cells can be manufactured as a homogeneous, off-the-shelf product for clinical use [46]. They can be administered topically, intramuscularly, or by intravenous administration as an advanced-therapy medicinal product (ATMP) [47]. An additional advantage of ABCB5^+^ MSCs over classic MSCs, such as ADSCs and BM-MSCs is that ABCB5^+^ MSCs can be easily obtained from discarded skin tissues donated primarily by individuals undergoing plastic surgery, without the need for ad hoc invasive procedures [46].

**Figure 2:**
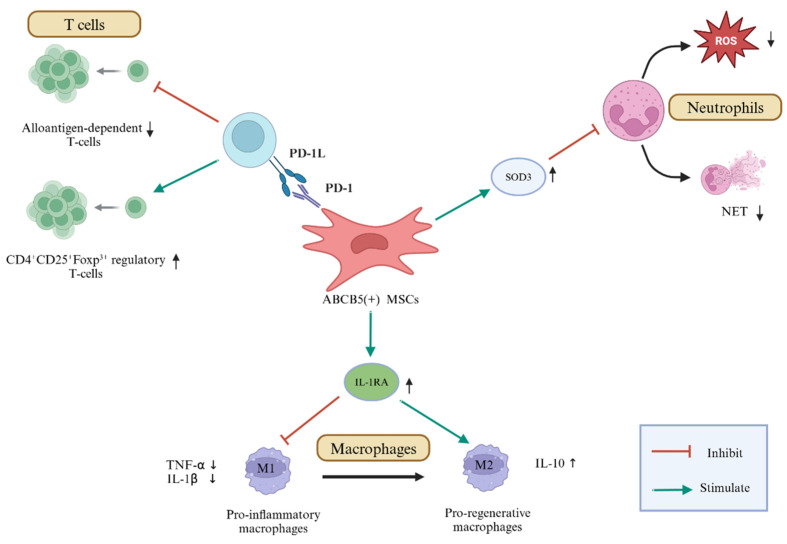
Immunomodulatory mechanisms of ABCB5^+^ MSCs.

Importantly, dermal ABCB5^+^ MSCs possess the ability to modulate immune function by interacting with immune cells including T-cells, neutrophils, and macrophages (Figure 2). ABCB5^+^ MSCs can evade immune rejection, home to the recipient immune tissues, and suppress alloantigen-dependent T-cell proliferation by interacting with programmed cell death 1 (PD-1) expressed on the ABCB5^+^ MSCs and PD-L1 expressed on the host cells. They also induce splenic CD4^+^CD25^+^Foxp^3+^ regulatory T-cells (Tregs) in vivo [41]. Additionally, ABCB5^+^ MSCs suppress the tissue damage caused by overactivated neutrophils through the adaptive release of superoxide dismutase 3 (SOD3), which reduces reactive oxygen species (ROS) levels in the microenvironment. This suppression prevents the formation of neutrophil extracellular traps (NETs), neutrophil death, and the excessive spillage of proteases [48]. Furthermore, ABCB5^+^ MSCs stimulate a phenotypic shift in macrophages from tumor necrosis factor-alpha (TNF-α)- and interleukin-1β (IL-1β)-secreting M1 pro-inflammatory macrophages to IL-10-secreting M2 pro-regenerative macrophages, mediated by the interleukin-1 receptor antagonist (IL-1RA) released from the stimulated ABCB5^+^ MSCs [40].

The trophic and exocrine functions of ABCB5^+^ MSCs are hallmark features of these cells. In response to inflammatory signals, ABCB5^+^ MSCs secrete IL-1RA and produce angiogenic growth factors such as the vascular endothelial growth factor (VEGF) and angiogenin (ANG), as well as basement membrane proteins including collagen type VII and laminin-322 [49,50,51]. Furthermore, upon short-term interaction with M1 macrophages, ABCB5^+^ MSCs upregulate the immune cell recruitment molecules, including chemokine (C-X-C motif) ligand (CXCL) family members CXCL2 and CXCL10, and interleukins including IL-1β and IL-6. This suggests that during the early stages of inflammation when macrophages are critical for countering pathogen invasion and clearing debris from injured sites, ABCB5^+^ MSCs may play a role in stimulating macrophage recruitment.

The angiogenic functions of ABCB5^+^ MSCs are largely mediated by VEGF secretion in response to hypoxic culture conditions through the activation of the hypoxia-inducible transcription factor 1α (HIF-1α) pathway [49,52]. In db/db mice models, the injection of ABCB5^+^ MSCs has been shown to promote wound healing by enhancing angiogenin release, which phosphorylates the downstream effector c-Src at Tyr416 position and pS6 ribosomal protein, thereby stimulating VEGFR2 signaling and enhancing growth signaling. Simultaneously, collagen deposition and Alpha-Smooth Muscle Actin (α-SMA) expression are induced, facilitating cellular matrix interaction and improving tissue repair and wound contraction, even in atrophic diabetic dermis [50].

## 3. Differentiation Potential of ABCB5^+^ MSCs

**Figure 3:**
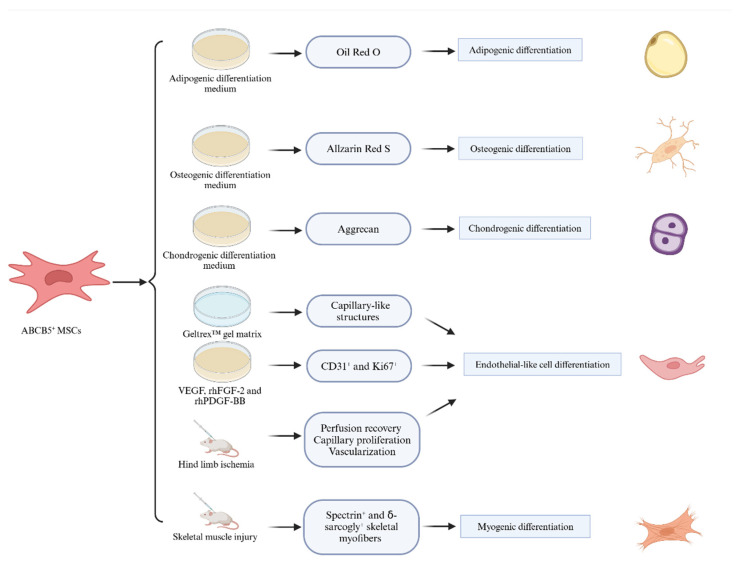
Differentiation potential of ABCB5^+^ MSCs.

The classic trilineage differentiation potential of MSCs includes adipocyte, chondrocyte, and osteocyte differentiation [40]. In addition to that, ABCB5^+^ MSCs demonstrate a myogenic differentiation potential [53]. Furthermore, ABCB5^+^ MSCs can acquire the phenotypic and functional characteristics of endothelial cells [49]. However, multiple attempts to induce hepatocytic lineage differentiation have been unsuccessful (Figure 3).

### 3.1. Classic Trilineage Differentiation

The trilineage differentiation ability, which includes adipogenic, osteogenic, and chondrogenic differentiation under specific differentiation conditions (Table 2), is one of the hallmark features of MSCs [42] and has also been demonstrated for ABCB5^+^ MSCs [40].

To further characterize the self-renewal capacity and differentiation potential of ABCB5^+^ MSCs, single cell-derived colony growth was assessed. The results showed that 75.61% of these colonies exhibited clonogenic growth. Of these, 62.40% retained their potential to differentiate into trilineage cells. Among those differentiable clones, 29.84% were bipotent, 7.77% were unipotent for osteogenic differentiation, and none were negative for all three lineages [40]. Interestingly, as donor age increased, ABCB5^+^ cells demonstrated an increased adipogenic differentiation capacity, which was accompanied by a reduced ability for chondrogenic and osteogenic differentiation [56].

### 3.2. Endothelial-like Cell Differentiation

The ability of MSCs to differentiate into endothelial cells holds significant promise for cardiovascular regeneration and angiogenesis [57,58]. ABCB5^+^ MSCs can differentiate into functional endothelial-like cells under special conditions. During an angiogenic trans-differentiation assay, induced with a basic dose of recombinant human (rh) VEGF, rhFGF-2, and rhPDGF-BB for 96 h, ABCB5^+^ MSCs exhibited the increasing expression of CD31 and Ki67, adopting the characteristics of proliferating endothelial cells. In contrast, undifferentiated ABCB5^+^ MSCs do not express CD31 [41,49].

Further evidence of endothelial differentiation was observed after the topical application with low, mid, and high doses of human-derived ABCB5^+^ MSCs to punch biopsy wounds in NOD.Cg-PrkdcscidIl2rgtm1Wjl/SzJ (NSG) mice, which have severe immunodeficiency. A dose-dependent increase in CD31 expression was detected, accompanied by a decrease in wound size, providing additional support for the endothelial differentiation capacity of ABCB5^+^ MSCs [59]. Additionally, endothelial-differentiated ABCB5^+^ MSCs formed capillary-like structures similar to those generated by human umbilical vein endothelial cells (HUVECs) when cultured on a Geltrex™ gel matrix [49].

Animal experiments have further corroborated these findings. In a hind limb ischemia model, the intramuscular injection of ABCB5^+^ MSCs into Oncins France 1 (OF1) mice (a strain characterized by rapid growth) resulted in increased perfusion recovery, enhanced capillary proliferation, and vascularization [49]. Under physiological conditions, human ABCB5^+^ MSCs are primarily located in a peri-vascular endogenous niche, where they are closely associated with CD31^+^ endothelial cells [40].

### 3.3. Myogenic Differentiation Potential

Adult stem cells and muscle satellite cells play a pivotal role in muscle regeneration [60,61]. When the natural regeneration capacity of skeletal muscle tissue is insufficient, it can lead to volumetric muscle loss (VML), which may result in chronic functional impairment and even functional disability. Therefore, the regeneration of myogenic tissue has become a focus of therapeutic interest. To date, MSCs derived from various tissues including adipose tissue, bone marrow, skeletal muscle, umbilical cord tissue, and urine have been differentiated into smooth muscle cells and skeletal muscle cells in vitro [62,63,64].

ABCB5^+^ MSCs have also been shown to form human spectrin- and δ-sarcogly-expressing skeletal myofibers, accelerating skeletal muscle regeneration in a mouse skeletal muscle injury model. This finding highlights their myogenic differentiation potential in vivo [53]. As ABCB5^+^ MSCs can be more easily derived from human tissues than other MSCs [51], studies are currently optimizing their ability to fully differentiate into myogenic tissues in vitro and in vivo.

### 3.4. Hepatocytic Differentiation Potential

In the search for liver replacement therapies, previous studies have explored whether MSCs can differentiate into cells of the hepatocytic lineage. An established single-step protocol [65], which has recently been used in other MSCs, was tested on ABCB5^+^ MSCs. While these cells failed to acquire the typical characteristics of hepatocytes after 14 days of incubation in the differentiation medium [66], the cells expressed thrombospondin 1, HGF, and monocyte chemoattractant protein 1 (MCP-1), all of which are involved in tissue remodeling and morphogenesis in the liver [66,67]. It remains to be seen if further cell culture protocols can induce clear hepatocytic differentiation.

## 4. The Therapeutic Potential of MSCs

The sources of MSCs-based therapy can be categorized into the following two types: (1) autologous, where cells are derived from patients themselves, defined as self-to-self therapy, and (2) allogeneic, where cells are isolated and expanded from healthy donors for the treatment. In general, MSCs exhibit low immunogenicity, allowing their application without the need for any immunosuppressive treatment in clinical settings [13,68]. This is particularly significant given the limited availability of resources. Autologous applications are often more time-consuming and costly for patients. In addition, both the limited quantity and poor quality of the MSCs obtained from older patients or those with systemic diseases, such as diabetes, significantly reduce the effectiveness of autogenous MSCs-based treatments.

In contrast, allogenic ABCB5^+^ MSCs, derived from rigorously selected donors and expanded to a ready-to-use scale with proven purity, potency, safety, and tolerability, hold the most promise for clinical applications.

## 5. Potential Clinical Applications of ABCB5^+^ MSCs

After more than 20 years of development, MSCs-based therapy has expanded into a wide range of clinical applications. As of 10 March 2024, over 1450 clinical trials involving MSCs have been registered in the public clinical trial database, http://clinicaltrials.gov. These trials address a variety of diseases affecting multiple systems and multiple organs. ADSCs, in particular, have been clinically applied in fields such as orthopedics, cardiology, and nephrology [17,20,69], and they also hold potential for angiogenesis [70]. Additionally, the clinical applications of ABCB5^+^ MSCs have been evaluated in several studies (Table 3).

### 5.1. Chronic Skin Wounds

Wound healing is influenced by multiple factors, including depth, location, patients’ ages, and comorbidities. Chronic wounds fail to progress through the normal, orderly sequence of repair required to restore normal tissue anatomy and function. These wounds are typically characterized by elevated levels of cytokines and proteases, which degrade essential ECM components, growth factors, and growth factor receptors [76].

MSCs have been shown to accelerate wound closure by reducing inflammation, enhancing angiogenesis, promoting re-epithelialization, and improving granulation [77,78]. Human skin-derived ABCB5^+^ MSCs exhibit skin-homing and engraftment properties [51]. These cells facilitate the transition of an M1 pro-inflammation macrophage to an M2 pro-regeneration macrophage, suppress ROS level and NET formation, and exert trophic effects involved in angiogenesis and ECM remodeling [40,48,49,51]. These findings highlight the potential therapeutic benefits of ABCB5^+^ MSCs in the treatment of chronic wounds.

#### 5.1.1. Chronic Venous Ulcers

Chronic venous ulcers (CVUs) account for approximately 75% of all leg wounds and typically develop in the medial lower leg. These ulcers are primarily caused by chronic venous insufficiency, which results from conditions such as varicose veins or post-thrombotic syndrome and leads to chronic venous hypertension in the affected tissue. Consequentially, hemosiderin, derived from extravasated erythrocytes, induces macrophages to shift into an iron-overloaded M1 pro-inflammatory state. These macrophages overexpress inflammatory mediators, including TNF-α and IL-1β, which exacerbate inflammation. Excessive oxidative stress, caused by increased levels of ROS, promotes cell senescence and apoptosis, contributing to tissue destruction and dermal disintegration. Furthermore, immune cells secrete matrix metalloproteinases (MMP), such as MMP-2 and MMP-9, which degrade the ECM and inhibit the progression to the proliferative stage of healing. Bacterial superinfection of the wound may further perpetuate the inflammatory phase [79,80]. ABCB5^+^ MSCs abrogate the M1 pro-inflammation state of macrophages and shift them into an M2 pro-regeneration state by secreting IL-1RA [40]. These cells are an option for the treatment of therapy-resistant chronic venous ulcers.

Most clinical modalities for treating venous ulcers target venous hypertension. However, there is a certain percentage of patients who fail to respond to standard treatment. In a first-in-human phase 1/2a clinical trial (NCT02742844), published in 2021 [59], investigated autologous ABCB5^+^ MSCs in 9 patients with 12 standard-treatment refractory CVUs (9 target and 3 non-target wounds). Wounds were debrided under local anesthesia, and a suspension of ABCB5^+^ MSCs containing 1 × 10^7^ cells/mL was applied to the wound surface at a concentration of 5 × 10^5^ cells/cm^2^ wound area. The wounds were immediately covered with Tegaderm film dressing, which was replaced by foam dressings after 1–3 days and maintained until week 12, along with the standard compression dressings. Of the nine target ulcers, six were eligible to determine treatment efficacy. By week 6, a median wound size reduction of 59% (range, 29–84%) was observed, increasing to 63% (range, 32–100%) by week 12. Patients reported improved wound quality and early pain relief, with no adverse events related to cell treatment during a 12-month safety follow-up. Furthermore, this trial first evaluated the efficacy and safety of autologous ABCB5^+^ MSCs. For this, the cells were expanded ex vivo following a strict standard, allowing them to generate a highly functional homogeneous cell population manufactured as an ATMP. The producer ensured the identity, vitality, viability, and potency of these cells as well as the purity and stability of manufactured pharmaceutical products at the same time [59].

Based on this clinical trial using autologous ABCB5^+^ MSCs, an interventional, multicenter, single-arm trial with allogeneic ABCB5^+^ MSCs was conducted (NCT03257098). The treatment involved the topical application of ABCB5^+^ MSCs at a dose of 1 × 10^6^ cells/cm^2^, followed by film dressing replacement with foam dressing after 1–3 days, alongside standard compression dressings. A total of 31 patients with therapy-refractory ulcers (wounds without decrease or increase above 25% after standard care) received treatment, with 22 patients undergoing two administrations 6 weeks apart and 9 patients receiving a single treatment. By week 12, 21 patients responded, achieving a median wound size reduction of 87%, with 29% (6 of 21) of wounds showing complete wound closure. Up to 3 treatment-emergent adverse events (TEAEs) related to the cell product were reported, including increased wound exudation, erythema, and venous ulcer pain, all of which were mild or moderate and recovered without sequelae [71]. A subsequent larger, randomized, placebo-controlled, double-blind phase 2b clinical trial with a dose-ranging design is ongoing (NCT04971161).

#### 5.1.2. Diabetic Foot Ulcer

Diabetic foot syndrome (DFS) is one detrimental complication of diabetics, resulting from poor glycemic control, peripheral vascular diseases, neuropathy, trauma, and impaired resistance to infection [81,82]. It often leads to recurrent infections, hospitalizations, gangrene, and, in severe cases, amputation thus imposing a significant financial burden on the health system. The potential capacity of MSCs in improving diabetic foot ulcer healing has gained considerable attention. Up to now, both bone marrow-derived MSCs and adipose-derived MSCs have been used as novel treatment options in diabetic foot ulcers in both preclinical and clinical trials [83].

In vitro, the peripheral vascular ischemia associated with diabetic patients can be simulated by hypoxia in cell culture chambers. Under hypoxia, ABCB5^+^ MSCs have been shown to secrete VEGF through the HIF-1α pathway, increasing the expression of endothelial-lineage marker CD31 and facilitating the formation of capillary-like structures on a gel matrix [49,52]. Furthermore, the injection of ABCB5^+^ MSCs at the edge of the non-healing wounds in the diabetic db/db mice enhanced angiogenesis and accelerated wound closure. These effects were significantly reduced when angiogenin was silenced in ABCB5^+^ MSCs before injection, indicating that ABCB5^+^ MSCs may play a critical role in the regenerative therapy of diabetic foot ulcers [50].

In a clinical trial (NCT03267784), one or two topical applications of ABCB5^+^ MSCs at a dose of 2 × 10^6^ cells/cm^2^ to therapy-refractory diabetic foot ulcers lead to a median wound surface area reduction of 59% (full analysis set, n  =  23), 64% (per-protocol set, n  =  20) and 67% (subgroup of responders, n  =  17) by week 12. Complete wound closure was achieved in six patients (26%, 30%, and 35% of patients in the full analysis set, per-protocol set, and a subgroup of responders, respectively). No treatment-related adverse events were observed [49].

#### 5.1.3. Recessive Dystrophic Epidermolysis Bullosa

Recessive dystrophic epidermolysis bullosa (RDEB) is a rare, inherited, life-threatening skin disease characterized by recurring, chronic non-healing wounds accompanied by pain and itching. The condition is caused by mutations in the Col7a1 gene, leading to a deficiency in functional collagen VII (C7). C7 promotes keratinocyte re-epithelization through interaction with laminin-332 in hemidesmosomes, supports fibroblast migration, and regulates cytokine production [84,85]. Systemic immunological defects in RDEB contribute to an intrinsic pro-inflammatory state with high levels of cytokines, such as IL-1β, IL-2, and IL-6. Extensive neutrophil infiltration and activation of CD38^+^ inflammatory macrophages further exacerbate tissue damage and chronic inflammation [86,87,88]. Although no curative therapy exists for RDEB, stem cell-based therapies may lead to wound regeneration thus alleviating symptoms [89].

Given their immunomodulatory properties and strong skin-homing ability, ABCB5^+^ MSCs are promising candidates for RDEB therapy [51]. To verify this in vivo, the influence of ABCB5^+^ MSCs on RDEB was evaluated in an immunodeficient Col7a1−/− mouse model of RDEB with blistered wounds. In this mouse strain, the clustered regularly interspaced short palindromic repeats and associated nuclease (CRISPR/Cas9) system were combined with microinjection into NOD/SCID IL2rγcnull (NSG) embryos to knock out the Col7a1−/− gene. These mice develop spontaneous blisters and wounds characteristic of skin fragility seen in RDEB [72]. Treatment with 5 × 10^5^ ABCB5^+^ MSCs in 10 µL volume via facial vein injection significantly improved general health status and survival compared to non-transplanted controls.

Webber and colleagues suggested that the therapeutic effect and enhanced survival could be attributed primarily to the immunomodulatory function of ABCB5^+^ MSC, which was supported by a significant reduction of skin infiltration with macrophages observed in the ABCB5^+^ MSC-treated Col7a1-knockout mice [72]. Another group compared ABCB5^+^ MSCs to BM-MSCs. After intravenous injection into NOD-scid IL2rγ null (NSG) mice with full-thickness dorsal skin wounds, ABCB5^+^ MSCs showed a better skin engraftment potential than BM-MSCs, which the authors attributed to an increased expression of HOXA3. ABCB5^+^ MSCs furthermore showed an expression of C7 while bone marrow-derived MSCs did not, even though they secrete the immunosuppressive IL-1RA [51].

In a phase 1/2a clinical trial (NCT03529877), 14 patients with RDEB received three intravenous infusions of 2 × 10⁶ ABCB5^+^ MSCs/kg body weight on days 0, 17, and 35. The treatment led to significant reductions in disease activity, itch, and pain scores [73]. A post-hoc analysis of 168 evaluable wounds that were present at baseline [74] showed that the therapy facilitated wound closure compared to historical placebo data. Approximately 75% of closed wounds remained permanently closed for at least 7 to 9.5 weeks, a period significantly longer than the typical recurrence interval of RDEB wounds, which averages around 3 weeks [75]. Similar outcomes were observed in a post-hoc analysis of 174 evaluable wounds that were newly developed over time [90]. Nearly half (44%) of these wounds occurred by day 17, while only 28% developed by day 35, and another 28% emerged over a longer 7-week period, indicating that ABCB5^+^ MSCs may delay the occurrence of new wounds. Moreover, the wounds that developed by day 17 exhibited a higher proportion of rapid healing (56% within 18 days) compared to baseline wounds (27% within 17 days). Of these early healing wounds, 88% remained stably closed for at least 7 weeks. These findings collectively support the application of ABCB5^+^ MSCs as a therapeutic option for symptomatic RDEB patients. A subsequent larger, randomized, placebo-controlled, double-blind phase 3 clinical trial is ongoing (NCT05464381).

### 5.2. Chronic Liver Disease

Chronic liver disease (CLD) can lead to fibrosis and cirrhosis, conditions characterized by ongoing inflammation, destruction, and regeneration of liver parenchyma, accompanied by a progressive deterioration of liver function. This decline impairs the liver’s ability to synthesize proteins (e.g., clotting factors), detoxify harmful substances, and excrete bile. The etiology of CLD is diverse and includes alcoholic liver disease, non-alcoholic fatty liver disease (NAFLD), liver toxins, autoimmune hepatitis, chronic viral hepatitis, and genetic disorders such as alpha-1 antitrypsin deficiency, hereditary hemochromatosis, and Wilson’s disease [91]. From a pathophysiological perspective, the inflammatory response in CLD triggers the activation of hepatic stellate cells (HSCs), which, in turn, leads to chemokine-mediated infiltration of immune cells, including macrophages, neutrophils, monocytes, natural killer (NK) cells, and natural killer T (NKT) cells. These immune cells collectively contribute to the progression of liver fibrosis [92,93].

When cultured in a hepatocytic differentiation medium, ABCB5^+^ cells secrete a variety of cytokines, chemokines, and growth factors, including vascular cell adhesion molecule 1 (VCAM-1), HGF, and MCP-1. These factors play key roles in tissue remodeling, morphogenesis, inflammation, and immune regulation in liver repair. These in vitro findings suggest that ABCB5^+^ MSCs may contribute to or even prompt hepatic morphogenesis during tissue remodeling after liver injury [66]. While BM-MSCs have been shown to differentiate into fibrogenic myofibroblasts and potentially exacerbate fibrosis [94], ABCB5^+^ MSCs demonstrated no such effects in the Mdr2KO mice, a model of induced liver fibrosis and partial liver resection. Injection of ABCB5^+^ MSCs into these mice did not result in liver damage, inflammation, or fibrosis. Moreover, there was no increase in pro-inflammatory cytokine expression, including IL1B and IL6, while tissue inhibitors of metalloproteinases 1 (TIMP1) showed a decreasing trend. Post-transplantation, there was no indication of toxicity, and the key health indicators such as body weight, liver-to-body weight ratio, and liver function parameters remained within the normal ranges, demonstrating a favorable safety profile. Additionally, ABCB5^+^ MSC treatment reduced collagen deposition and decreased the number of activated HSCs, further supporting its anti-fibrotic potential [67].

Taken together, these findings suggest that ABCB5^+^ MSCs may carry therapeutic potential for chronic liver diseases by attenuating fibrosis and promoting functional recovery through their immunomodulatory and trophic effects. However, further in vivo and in vitro studies are required to confirm and expand upon these findings.

### 5.3. Graft-Versus-Host Disease

GvHD is a serious complication of allogeneic hematopoietic stem cell transplantation (HSCT), characterized by a wide range of symptoms, including skin rash, gastrointestinal dysfunction, and cholestatic liver disease. GvHD arises from a systemic cytotoxic attack by alloantigen-specific donor T cells delivered with the graft [95,96,97]. The progression of GvHD typically occurs in three distinct steps. First, tissue damage caused by pre-transplant conditioning regimens and/or concurrent inflammatory processes triggers the secretion of pro-inflammatory cytokines, such as TNF-α and IL-1, which activate the host antigen-presenting cells (APCs). Second, following HSCT, these activated APCs cross-present host antigens to donor T cells, leading to their allo-activation and proliferation. Finally, cytotoxic donor T lymphocytes (CTLs) directly damage tissues through the induction of apoptosis and necrosis [98].

ABCB5^+^ MSCs have demonstrated the ability to suppress T-cell proliferation, evade immune rejection, home to the recipient immune tissues, and induce Tregs in vivo through interactions between PD-1 expressed on ABCB5^+^ MSCs and PD-L1 expressed on the host cells. These properties suggest that ABCB5^+^ MSCs may hold significant potential in treating GvHD [41]. Additionally, ABCB5^+^ MSCs have been shown to modulate the immune response by reducing the secretion of TNF-α and IL-2, two cytokines critical in the development of GvHD, while concurrently increasing IL-10, a cytokine essential for limiting GvHD progression [40,99,100,101]. However, further evidence is needed to confirm the safety and efficacy of ABCB5^+^ MSCs in potential clinical applications.

## 6. Discussion and Clinical Perspectives

The differentiation ability of ABCB5^+^ MSCs has been demonstrated across multiple experimental settings, highlighting their potential in tissue regeneration. Research into their ability to differentiate into endothelial and myogenic cells, alongside optimizing differentiation protocols, could uncover critical underlying mechanisms and broaden clinical applications in regenerative medicine. Recently, the following two distinct types of ABCB5^+^ stem cells have been identified: human skin-derived ABCB5^+^ MSCs and ABCB5^+^ limbal stem cells [102,103]. The differentiation potential of these two cell types remains an area of great interest and warrants further investigation. In particular, the potential application of ABCB5^+^ stem cells in ophthalmology is especially promising. For instance, investigating whether and how ABCB5^+^ MSCs can differentiate into corneal cells could pave the way for innovative treatments for ocular diseases.

Traditionally, cell experiments have relied on 2D culture models, which fail to capture the complexity of human physiology. The emergence of 3D culture models provides a more accurate representation of physiological and pathological conditions, providing valuable insights into the immunomodulatory and regenerative properties of MSCs. Applying these advanced models may further characterize the unique properties of ABCB5^+^ MSCs [104,105].

Although ABCB5 has been extensively studied in cancer biology, emerging evidence suggests that it is also expressed by normal cells [106]. The prospect of MSCs-based therapies in cancer is another fascinating facet of cutting-edge research. As a transmembrane P-glycoprotein regulating the efflux of substances as an MDR transporter, ABCB5 regulates the efflux of various substances, potentially enabling skin-derived ABCB5^+^ MSCs to interfere with drug resistance in cancers such as melanoma, thereby enhancing the efficacy of chemotherapy.

Since MSCs are extensively studied and applied in clinical treatment, like regenerative medicine, it is crucial to ensure their long-term safety, therapeutic efficacy, and close monitoring for any toxicities. Reports on traditional MSCs, including ADSCs and BM-MSCs, have shown promising results across various conditions, demonstrating the safety, effectiveness, and tolerability of MSC-based therapies. Similarly, clinical data on ABCB5^+^ MSCs have also confirmed their efficacy, stability, and safety. Nonetheless, extended long-term studies are necessary to further validate these findings and ensure consistent outcomes.

To summarize, ABCB5^+^ MSCs represent a novel and promising lineage of stem cells that can currently be produced through rigorous manufacturing processes that ensure homogeneity, potency, and safety. While ongoing research continues to validate their efficacy and expand their clinical applicability, their unique properties and demonstrated therapeutic potential already make them valuable tools in regenerative medicine and clinical treatments.

Building on this foundation, ABCB5^+^ MSCs hold great promise for broader clinical applications in the future. Future research could investigate their roles in cardiovascular regeneration, hepatic remodeling, autoimmune and non-autoimmune diseases, cancer therapy and, as mentioned, ocular diseases. Emerging technologies, such as genetic engineering, may unlock new possibilities for optimizing their differentiation, expansion, and therapeutic efficacy. For example, their skin-homing ability, unique immunomodulatory properties, and multilineage differentiation capabilities make them ideal candidates for integration with gene therapy. By utilizing tools like CRISPR/Cas9, ABCB5^+^ MSCs can serve as carriers for therapeutic genes, addressing genetic disorders, such as hereditary skin diseases and cancers. Additionally, their potential to enhance the engraftment and long-term survival of gene-edited cells could significantly improve the stability and effectiveness of gene therapies.

In postoperative recovery, ABCB5^+^ MSCs may accelerate tissue regeneration and minimize inflammation by secreting angiogenic factors like VEGF and promoting the phenotypic shift of macrophages from the pro-inflammatory state to the pro-regenerative state. These properties provide them with an essential role in complex surgical procedures, such as vascular reconstructive surgeries and organ transplants, where improved healing and reduced immune rejection are crucial. With continued advancements in research and clinical applications, ABCB5^+^ MSCs hold the potential to address unmet clinical needs across diverse fields of medicine.

## Figures and Tables

**Table 1:** Potential clinical therapeutic application of human mesenchymal stem cells.

MSC Type	Resource	Investigated as Potential Therapeutic Targets	Citation
ADSCs	Adipose tissue	chronic ischemic cardiomyopathy, Sjogren’s syndrome, chronic kidney disease, ovarian cancer, knee osteoarthritis	[15,16,17,18,19,20]
BM-MSCs	Bone marrow	GvHD, aplastic anemia, Parkinson’s Disease, pulmonary fibrosis, Chronic Patellar Tendinopathy	[21,22,23,24,25]
UC-MSCs	Umbilical cord	Psoriasis, peripheral arterial disease, liver cirrhosis, skin scars, knee osteoarthritis	[26,27,28,29,30]
Placenta-derived MSCs	Placenta	Perianal fistulae, knee osteoarthritis	[27,31]
DPSCs	Dental pulp	Dental pulp regeneration, intrabony defects, acute ischemic stroke	[32,33,34]

ADSCs: adipose tissue-derived mesenchymal stem cells; GvHD: graft-versus-host disease; UC-MSCs: umbilical cord-derived mesenchymal stem cells; DPSCs: dental pulp stem cells.

**Table 2:** Classic trilineage differentiation conditions of MSCs.

Differentiation Type	Conditions	Citation
Adipogenic differentiation	High glucose DMEM, 10% FBS, 1 μm dexamethasone, 200 μm indomethacin, 10 μg/mL insulin, 0.5 mm methylisobutyxanthine	[54]
Osteogenic differentiation	High glucose DMEM, 10% FBS, 50 μM ascorbic acid-2-phosphate, 10 mm β–glycerophosphate and 100 nm dexamethasone	[54]
Chondrogenic differentiation	High Glucose DMEM, 10% FBS, 10^−7^ M dexamethasone, 1 μM ascorbate-2-phosphate, 1% sodium pyruvate, and 10 ng/mL transforming growth factor-beta 1 (TGF-β1).	[55]

**Table 3:** Summary of literature on the (potential) clinical applications of ABCB5^+^ MSCs.

Clinical Application	Study Type	Reference
Chronic venous ulcer	Clinical (Human)	[59,71]
Diabetic foot ulcer	Preclinical and Clinical	[49,50]
Recessive dystrophic epidermolysis bullosa	Preclinical and Clinical	[72,73,74,75]
Chronic liver disease	Preclinical (Animal)	[66,67]
Graft-versus-Host Disease	Preclinical (Animal)	[41]

## Abbreviations

MSCs	Mesenchymal stem cells
ADSCs	Adipose tissue-derived mesenchymal stem cells
BM-MSCs	Bone marrow-derived MSCs
UC-MSCs	Umbilical cord-derived mesenchymal stem cells
ABCB5^+^ MSCs	ATP-binding cassette subfamily B member 5 positive MSCs
ECM	Extracellular Matrix
GvHD	Graft-versus-host disease
DPSCs	Dental pulp stem cells
iPSC	Induced pluripotent stem cell
MDR	Multidrug resistance
ATMP	Advanced-therapy medicinal product
PD -1	Programmed cell death 1
Tregs	Regulatory T-cells
SOD3	Superoxide dismutase 3
ROS	Reactive oxygen species
NET	Neutrophil extracellular traps
TNF-α	Tumor necrosis factor-alpha
IL-1β	Interleukin-1β
IL-1RA	Interleukin-1 receptor antagonist
VEGF	Vascular endothelial growth factor
PDGF	Platelet-derived growth factor
ANG	Angiogenin
HGF	Hepatocyte growth factor
FGF	Fibroblast growth factor
CXCL	Chemokine (C-X-C motif) ligand
HIF-1α	Hypoxia-inducible transcription factor 1α
α-SMA	Alpha-Smooth Muscle Actin
TGF-β1	Transforming growth factor-beta 1
HUVECs	Human umbilical vein endothelial cells
OF1	Oncins France 1
VML	Volumetric Muscle Loss
MCP-1	Monocyte chemoattractant protein 1
CVU	Chronic venous ulcers
MMP	Matrix metalloproteinases
TEAEs	Treatment-emergent adverse events
DFS	Diabetic foot syndrome
SPs	Stromal precursors
RDEB	Recessive dystrophic epidermolysis bullosa
CRISPR/Cas9	Clustered regularly interspaced short palindromic repeats
C7	Collagen VII
CLD	Chronic liver disease
NAFLD	Non-alcoholic fatty liver disease
HSCs	Hepatic stellate cells
NK	Natural killer
NKT	Natural killer T
VCAM-1	Vascular cell adhesion molecule 1
TIMP1	Tissue inhibitors of metalloproteinases 1
HSCT	Hematopoietic stem cell transplantation
APCs	Antigen-presenting cells
CTLs	Cytotoxic T lymphocytes
